# Stochastic simulation algorithms for Interacting Particle Systems

**DOI:** 10.1371/journal.pone.0247046

**Published:** 2021-03-02

**Authors:** Timothy C. Stutz, Alfonso Landeros, Jason Xu, Janet S. Sinsheimer, Mary Sehl, Kenneth Lange

**Affiliations:** 1 Department of Computational Medicine, University of California, Los Angeles, CA, United States of America; 2 Department of Statistical Sciences, Duke University, Durham, NC, United States of America; 3 Department of Human Genetics, David Geffen School of Medicine, University of California, Los Angeles, CA, United States of America; 4 Department of Biostatistics, UCLA Fielding School of Public Health, Los Angeles, CA, United States of America; 5 Division of Hematology-Oncology, Department of Medicine, David Geffen School of Medicine, University of California, Los Angeles, CA, United States of America; 6 Department of Statistics, University of California, Los Angeles, CA, United States of America; University of Edinburgh, UNITED KINGDOM

## Abstract

Interacting Particle Systems (IPSs) are used to model spatio-temporal stochastic systems in many disparate areas of science. We design an algorithmic framework that reduces IPS simulation to simulation of well-mixed Chemical Reaction Networks (CRNs). This framework minimizes the number of associated reaction channels and decouples the computational cost of the simulations from the size of the lattice. Decoupling allows our software to make use of a wide class of techniques typically reserved for well-mixed CRNs. We implement the direct stochastic simulation algorithm in the open source programming language Julia. We also apply our algorithms to several complex spatial stochastic phenomena. including a rock-paper-scissors game, cancer growth in response to immunotherapy, and lipid oxidation dynamics. Our approach aids in standardizing mathematical models and in generating hypotheses based on concrete mechanistic behavior across a wide range of observed spatial phenomena.

## Introduction

Stochastic effects are crucial for accurately modeling evolutionary and biological processes such as tumor growth, desertification, disease spread, embryonic development, maintenance of species biodiversity, and pattern formation in general [1–3]. The associated spatial mathematical models are commonly analytically intractable. Fortunately the advent of efficient computing has allowed simulation to serve as a common first approach to stochastic modeling. Non-spatial well-mixed versions of these models are often substituted due to their tractability and ease of use. Many celebrated simulation algorithms such as the exact Stochastic Simulation Algorithm (SSA), *τ*-leaping, and the next-reaction method have been developed and extensively modified to address a wide range of well-mixed stochastic phenomena [4]. However, well-mixed models fail to capture the appropriate statistics and pattern formation seen in the spatial setting. Phenomena due to volume exclusion and spatial dispersion cannot be accurately captured via well-mixed Chemical Reaction Network (CRN) simulation. One common approach to stochastic spatial simulation is to partition the spatial domain into well-mixed voxels. This approach utilizes a Reaction-Diffusion Master Equation (RDME) to model the movement of particles between voxels and the reaction of particles within the same voxel. While this method has substantial algorithmic development [5], it fails to take into account important volume exclusion effects and fine-grained spatial variation. In particular, volume-exclusion has been shown to alter the mass-action kinetics observed in well-mixed models, instead producing fractal kinetics [6, 7]. This deviation from mass-action kinetics increases depending on the regularity of the spatial structure in question; for our lattice-based models, we expect to see significant departures from the well-mixed case due to these volume-excluding effects [6].

Interacting Particle Systems (IPSs) provide an alternative to both well-mixed CRN and RDME based modeling. IPSs are a class of stochastic models with full spatial detail, tracking each particle’s location on a lattice [8]. Interactions are assumed to be local, meaning particles must be adjacent to each other to interact. Notions of locality and adjacency are details that must be specified in a given model. For some typical reactions, see Table 1. Importantly, IPSs preserve volume exclusion, meaning at most one particle can be present on any given lattice site. Diffusive movement is typically modeled as particles undergoing random walks between sites, respecting exclusion. This is in contrast with more common RDME approaches that couple compartments obeying well-mixed dynamics through non-excluding Brownian motion. Recent significant advances in the basic RDME approach incorporate volume-excluding effects. These include the excluded volume reaction-diffusion master equation (vRDME) [9] and an approach that uses scaled particle theory to allow different-sized particles to have different diffusion rates between voxels [10]. Performing a full comparison between the different IPS and RDME approaches, volume-excluding and otherwise, is beyond the scope of this article. For a detailed modern review that explores the wide array of RDME and Brownian motion approaches to spatial stochastic simulation, we particularly recommend [11].

**Table 1:** Example processes with reaction diagrams.

Type	Example reactions	Processes
On-site	∅ → *A*	Immigration
	*A* → ∅	Death
	*A* → *B*	Transformation
Pairwise	*A* + ∅ → *A* + ∅	Migration
	*A* + ∅ → *A* + *A*	Binary fission
	*A* + *A* → *B* + ∅	Dimerization
	*A* + *B* → *C* + *D*	Pairwise transformation

∅ denotes an open site that is part of a reaction.

Example IPSs include the voter and contact processes as well as the classic Ising model from statistical mechanics [12]. These specific models have a large body of theoretical results from the mathematics community, specifically on their critical behavior. Unfortunately these results do not readily extend to multi-type processes and complicated spatial domains. Numerical approaches are computationally prohibitive, leaving direct simulation as the first and frequently only line of attack. The recent IPS simulation package Spatiocyte [13] and its numerous extensions address simulation biases generated under a lattice-based spatial structure [14] and parallelize the original simulation code [15]. We provide a more detailed description of the differences between our approach and Spatiocyte in the section “Availability and Future Directions.”

The current paper extends the classic *n*-fold simulation method, defined later, to IPSs [16]. Our extension enjoys three major advantages over previous approaches. First, we generate the minimum number of required reaction channels for a simulation, avoiding the combinatorial difficulties that arise from counting adjacent configurations of particles. Second, we provide efficient local updates after a reaction channel fires; thus only particles adjacent to a reaction are updated. Critically, this prevents the computational complexity of the simulations from scaling with the size of the lattice. Third, and perhaps most important, we separate the time and reaction sampling steps from the configuration update steps in the algorithm. This reduces our spatial process to the computational complexity of a CRN simulation, albeit with an additional complicated update. Accordingly, we can implement any CRN sampling algorithm for our spatial setting with little additional effort. Well-mixed CRN simulation is extensively developed [17–23]; therefore, spatial IPSs directly benefit from these prior innovations.

We build on the software package BioSimulator [24], written in the open source programming language Julia [25]. BioSimulator implements different algorithms for simulating IPSs, including the direct stochastic simulation algorithm (SSA) and versions of the next reaction method [26] and the sorting direct method [27]. Our software provides a simple, intuitive interface through which nonspecialists can quickly observe complex behaviors of spatial models with multiple interacting species. Summary statistics and particle count trajectories permit straightforward model checking for the proposed systems. Within this framework, modelers can determine which reactions and parameters are important for producing a certain desired behavior. A recent example of an IPS in action has been reported in a recent immunotherapy model for cancer treatment [28]. This complex model of tumor-immune system interactions illustrates which parameters generate the appropriate immune responses and spatial patterns.

Our software is primarily directed at systems biologists, cancer researchers, ecologists, evolutionary biologists, epidemiologists, and other scientists who are interested in the spatio-temporal effects of discrete actors. We anticipate that BioSimulator’s ease of use and flexibility will encourage researchers unfamiliar with stochastic processes to investigate the stochastic and spatial features of their models via simulation. Finally, our software allows users to avoid tedious re-implementation of different algorithms in their simulation studies.

The remaining exposition is organized as follows. First we give a mathematical description of IPSs. We then enumerate the different sample classes for probabilistically equivalent particles using the species types and neighborhood configurations of the lattice or graph. This enumeration plus a description of the reaction rates across these sample classes provides a straightforward means of extending the well-mixed SSA to IPSs. Lastly, we summarize how our software implements each reaction, including updating the sample classes and reaction rates. This is followed by a series of examples of complex, multi-species spatial stochastic phenomena. We conclude with a brief description of the benefits of writing BioSimulator in the Julia programming language.

## Design and implementation

### IPSs and pairwise reactions

An IPS models a collection of particles moving and reacting stochastically over some spatial domain. Particles are discrete entities that may model animals, proteins, wildfire patches, or cancer cells. Like well-mixed CRNs, these particles interact through a series of reaction channels. While stochastic CRNs assume every particle interacts uniformly with every other particle, IPSs restrict these interactions to neighboring particles. Each IPS has an associated graph describing the spatial domain over which the process evolves. Nodes on the graph are sites that a particle may occupy. Edges specify that two nodes are adjacent and hence liable to interact. Typically we restrict nodes to contain at most one particle at a time; we refer to this effect as *volume exclusion*.

Fortunately, embedding the IPSs on a graph allows us to restrict the reactions to being *pairwise*. We use the term pairwise instead of bimolecular deliberately; unimolecular reactions that produce two product particles require an open adjacent site due to the volume excluding effect. For example, birth through binary fission is written in well mixed reaction notation as *A* → *A* + *A*. On a graph with exclusion, birth requires an open adjacent site and becomes *A* + ∅ → *A* + *A* where ∅ denotes an open site that becomes occupied by one of the offspring particles. This schema emphasizes volume exclusion since birth cannot occur when the *A* particle has no open adjacent sites. We classify reactions into two groups, on-site and pairwise. For a non-exhaustive list of examples, see Table 1; for a specific predator-prey example see Table 2. These two reaction types, on-site and pairwise, are useful in describing a number of biological applications, but like all models they have their limitations when the system’s dynamics are complex. Higher-order reactions are reduced to pairwise interactions through the formation of intermediate complexes.

**Table 2:** Predator-prey reactions.

Name	Diagram	Type	Name	Diagram	Type
Fox Predation	*F* + *R* → *F* + *F*	Pairwise	Rabbit Reproduction	*R* + ∅ → *R* + *R*	Pairwise
Fox Migration	*F* + ∅ → ∅ + *F*	Pairwise	Rabbit Migration	*R* + ∅ → ∅ + *R*	Pairwise
Fox Death	*F* → ∅	On-site	Rabbit Death	*R* → ∅	On-site

Foxes (*F*) and rabbits (*R*) interact on a 2D hexagonal lattice with open sites (∅). Reactions are either on-site involving a single animal interacting only with itself, or pairwise involving an animal interacting with an adjacent site.

### Markovian dynamics, reaction channels, and sample classes

Particles evolve on the graph according to standard Markovian dynamics where the waiting time to the next reaction is exponentially distributed [29]. If a particle can take part in multiple reactions, then its exponential waiting time has rate equal to the sum of the rates of each individual reaction under mass-action kinetics. Note that more complicated kinetics are allowed provided that we restrict the interactions to neighboring particles. Longer range interactions are feasible in principle, though they introduce combinatorial complexity in enumerating the neighboring configurations. The current version of BioSimulator is restricted to mass-action kinetics for immediate neighbors.

**Fig 1:**
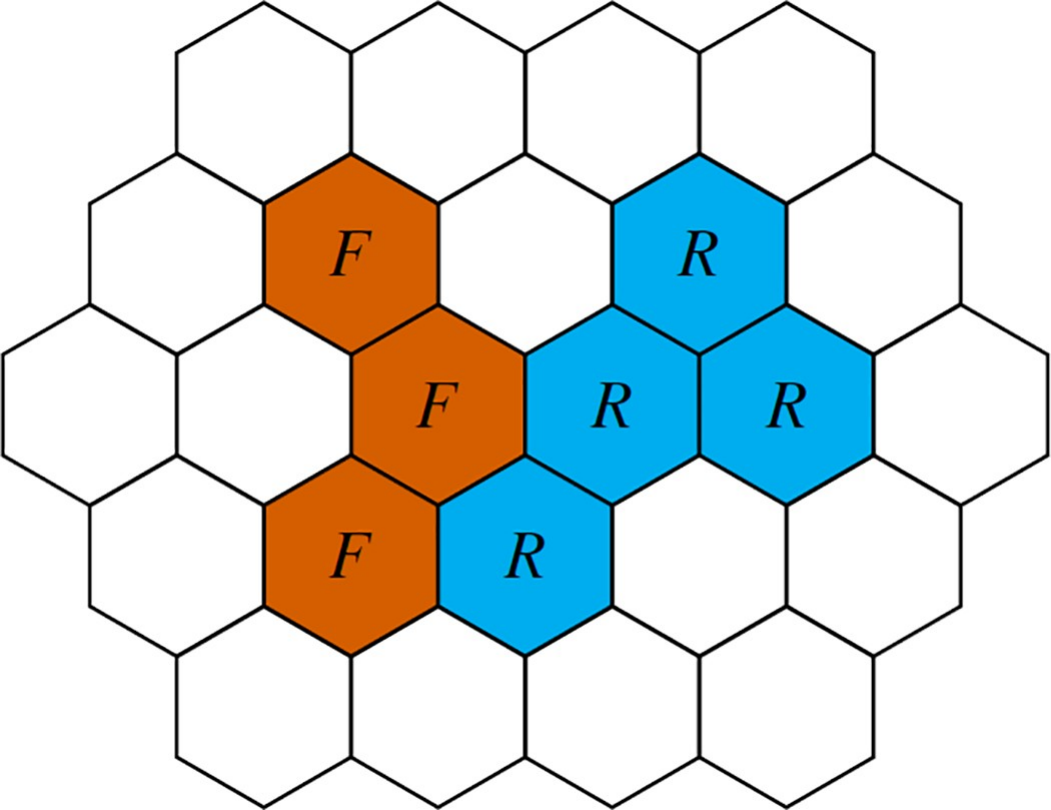
Example initial configuration. Sites are color coded by occupancy; vermvermillion denotes a fox *F*, and cyan denotes a rabbit *R*. A site can be occupied by at most one animal at a time. Open sites are left blank.

The rate at which a particle undergoes reactions depends on both the species of the particle and the number and species of its neighboring particles. Although open sites are not collectively considered a species, open sites next to occupied sites play a negative role in volume exclusion. In order to draw parallels with well-mixed CRNs, we split each pairwise reaction into a series of *reaction channels*. Each pairwise reaction channel is associated with a center particle interacting with up to *D* neighboring particles of the appropriate type, where *D* is the number of adjacent neighbors. *D* takes the values 4, 6, and 8, respectively, on a square planar lattice, a hexagonal planar lattice, and 3-dimensional cubic lattice. Therefore the total number of reaction channels is *R* = *D* × # pairwise reactions + # on-site reactions. See Fig 1 for a depiction of a predator-prey process involving foxes and rabbits on a hexagonal lattice and Table 3 for its associated reaction channels. For instance, when the third predation reaction channel fires, the simulation searches for a fox adjacent to exactly three rabbits to undergo the predation.

**Table 3:** Reaction channels and associated sample indices.

Reactants		Products		Per Particle Rate	Sample Index	Reactants		Products		Per Particle Rate	Sample Index
*F*	*R*	*F*	*F*	*α*	1	*F*	∅	∅	*F*	2*γ*	14
*F*	*R*	*F*	*F*	2*α*	2	*F*	∅	∅	*F*	3*γ*	15
*F*	*R*	*F*	*F*	3*α*	3	*F*	∅	∅	*F*	4*γ*	16
*F*	*R*	*F*	*F*	4*α*	4	*F*	∅	∅	*F*	5*γ*	17
*F*	*R*	*F*	*F*	5*α*	5	*F*	∅	∅	*F*	6*γ*	18
*F*	*R*	*F*	*F*	6*α*	6	*R*	∅	∅	*R*	*γ*	7
*R*	∅	*R*	*R*	*β*	7	*R*	∅	∅	*R*	2*γ*	8
*R*	∅	*R*	*R*	2*β*	8	*R*	∅	∅	*R*	3*γ*	9
*R*	∅	*R*	*R*	3*β*	9	*R*	∅	∅	*R*	4*γ*	10
*R*	∅	*R*	*R*	4*β*	10	*R*	∅	∅	*R*	5*γ*	11
*R*	∅	*R*	*R*	5*β*	11	*R*	∅	∅	*R*	6*γ*	12
*R*	∅	*R*	*R*	6*β*	12	*F*		∅		*μ*	19
*F*	∅	∅	*F*	*γ*	13	*R*		∅		*μ*	20

Each initial pairwise reaction in Fig 1a is split into six reaction channels, one for each number of adjacent reactants. Each reaction channel has an associated per particle rate and sample index. This sample index points to the collection of particles that the reaction channel samples a reactant from. The total rate of each reaction channel is equal to the per animal rate times the number of animals in the associated sample class. Note that the rabbit reproduction and migration channels share the same sample indices because they share the same reactants.

There are two approaches to sampling a reaction channel and associated particle. The more rudimentary approach is to scan through the particles in the lattice, sum the per-particle reaction rates, and select a particular particle to fire with probability proportional to its contribution to this sum [30]. A more sophisticated method is given by Bortz, Kalos, and Lebowitz under the *n*-fold way [16]. Here particles are grouped into classes such that all members of a given class take part in a specific reaction with the same rate. This explicitly forms a series of reaction channels for sampling using Markovian dynamics and avoids time consuming searches of the lattice.

We provide an extension to the *n*-fold way that decouples the sampling of the reaction channels, an inherently non-spatial maneuver, from the sampling of a particle to undergo the reaction. This in turn separates the spatial dependencies inherent in IPSs from the Markovian dynamics of the reaction channels. Thus, spatial correlations are handled during the update step. We do this via generating *sample classes*, which are collections of particles that can be sampled by one or more reaction channels. The sample classes are motivated by the observation that the exact configuration of neighboring particles does not matter for a given reaction channel firing. Only the number of neighboring particles of the appropriate type influence the reaction rate. Therefore each sample class contains particles of a specific species that are adjacent to a specific number of particles of a type that the particle under consideration can react with.

This is best demonstrated by an example; see the sample classes associated with each reaction channel in Table 3. The rabbits in the predator-prey example are sorted into seven different sample classes, numbers 7 through 12 and 20, one for each central rabbit interacting with one to six open adjacent sites and a final class containing only rabbits. As an example for the pairwise reaction channels, sample class 9 contains rabbits adjacent to three open sites. This sample class is targeted by two different reaction channels, one for rabbit migration with three neighbors and one for rabbit reproduction with three neighbors. Likewise there is a sample class associated with each on-site reaction; sample class 20 contains every rabbit that can undergo death.

Because multiple reaction channels may sample particles from the same sample class, the total number of sample classes is less than or equal to the number of reaction channels. Specifically, the number of sample classes is equal to *D* × # unique pairs of reactants + # unique on-site reactants. Using the list of reactants, we assign each reaction channel to its appropriate sample class. Multiple reaction channels will map to the same sample class when the reactions use the same pair of reactants. For example rabbit migration and reproduction map to the same sample class, as shown in Table 3.

### Local updates

For a reaction channel to fire, a particle is sampled uniformly from the appropriate sample class. This particle, possibly along with a neighbor of the appropriate interacting species, then undergoes the reaction. At this point, the reaction rates must be updated to reflect the changing configuration. Again there are two possible methods for updating these rates [30]. The first, called a *global update*, scans the entire lattice grouping particles into classes and calculating reaction rates. While straightforward, this is inefficient due to the fact that configuration changes take place over at most two neighboring particles. In contrast, we use a *local update* that changes the rates associated with particles immediately adjacent to or involved in the reaction. There is overhead associated with sorting the particles by class and the local updates, but these improvements prevent the simulation step from scaling with the number of particles in computational complexity.

**Fig 2:**
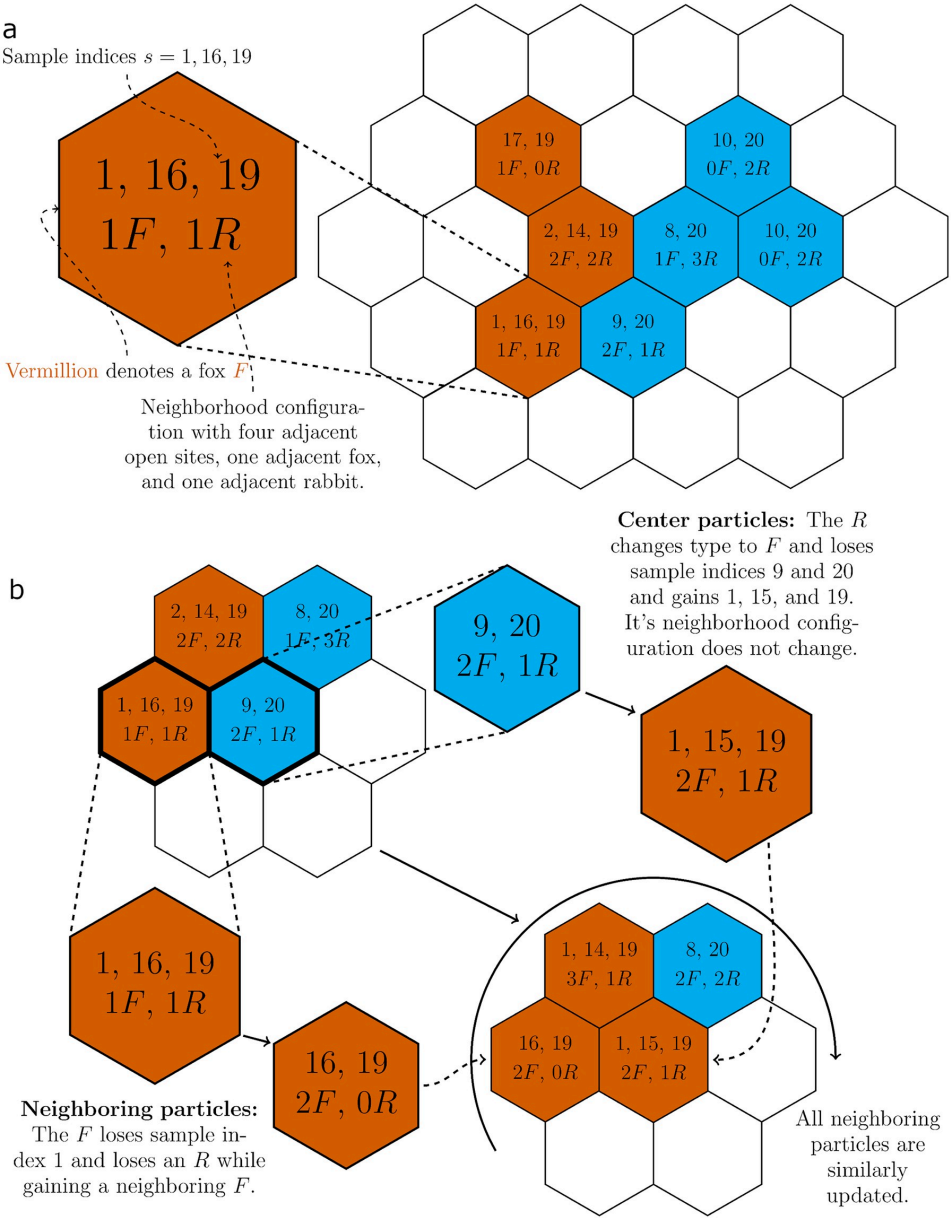
Configurations and reaction updating with sample classes. (a) Initial configuration with sample indices and neighborhood configurations. Note that the number of adjacent open sites can be inferred from the number of foxes and rabbits. (b) Updating the neighborhood and sample indices after a reaction. Suppose the highlighted fox and rabbit sites undergo a predation event. The rabbit is replaced with a fox, and so the neighborhood and sample indices of the sites surrounding the former *R* require updating to reflect the new configuration. The sample indices of the new *F* will change as well, but its neighborhood index will not. This update procedure need only be done for and around sites that change species.

We now expand upon how we perform local updates. Because each particle’s behavior depends only on its adjacent particles, it suffices to enumerate these different neighborhood configurations. Specifically, we count the number of ways *L* species can be distributed across *D* neighboring sites. The standard stars-and-bars argument shows that the total number of configurations *K* is equal to the binomial coefficient $\left(\begin{array}{c} D+L\\ L \end{array}\right)$. Highly efficient algorithms exist to systematically enumerate all configurations [31]. For example, with *D* = 4 neighbors and *L* = 2 species, the configuration 1 + 1 + 2 corresponds to one open adjacent site, one adjacent particle of the first type, and two adjacent particles of the second type. See Fig 2a for an example predator-prey model using the neighborhood configurations.

The naive approach to sampling particles for each reaction channel would be to group particles together by species and neighborhood configuration *k* ∈ {1, 2, …, *K*}. However, *K* scales factorially with the number of species in the simulation. This scaling issue further motivates our previous discussion of the sample classes, which scale with the number of reaction channels. We therefore restrict the use of neighborhood configurations purely for updating the sample classes after a reaction has occurred. We will now expand on how the neighborhood configurations, sample classes, and reaction channels interact.

Fig 2b provides an example of the local update procedure after a reaction channel has been chosen to fire. In this scenario, reaction channel 1 is firing, meaning the simulation searches for a fox *F* adjacent to exactly one rabbit *R* to undergo the predation reaction. Foxes that satisfy this condition are contained within sample class 1 as denoted in Table 3. Suppose the bolded fox in the first configuration of Fig 2b is sampled from sample class 1 to undergo the reaction. Since it has only one adjacent rabbit, also bolded, this rabbit is likewise sampled to be the target of the predation reaction. At this point the rabbit changes type to a fox, shifting from vermillion to cyan. The neighboring indices and sample classes of both particles and their adjacent species now are updated to reflect the rabbit changing type. For example, the sampled fox loses an adjacent rabbit and is removed from sample class 1 to reflect this change. Lastly we update the rates of the reaction channels that have changed in terms of numbers of associated particles.

This approach has two major benefits. First, it decouples the size of the simulation from the size of the lattice. The most intensive operations required are those involving sampling a particle in a given sample class. These operations scale *O*(*n*) with the number of elements *n* in the sample class. Second, this decouples the sampling algorithm from the update step, allowing us to extend our approach to arbitrary simulation algorithms. It is worth noting that a significant portion of the simulation time is spent updating the sample classes of a particle after a reaction has occurred; this is an unavoidable consequence of the spatial structure imposed by the lattice. See the first table in the S1 File for an example breakdown of the run-time of different parts of the simulation step.

### Extension to arbitrary simulation algorithms

We begin by demonstrating how our IPS sampling method maps neatly onto the SSA. Let *r* = 1, 2, …, *R* denote the index of a reaction channel, and let λ_*r*_ denote the associated reaction rate for the *r*-th reaction channel. λ_0_ = ∑_*r*_ λ_*r*_ is the total reaction rate for the process. Given *U*_1_ and *U*_2_ independent uniform [0, 1] random variables, we determine the time *T* to the next reaction and the next reaction channel *j* to fire by the conditions

$$\begin{array}{c} T=-\frac{\text{log}\;(U_{1})}{\lambda_{0}}\;\text{and}\;\sum_{r=1}^{j-1}\lambda_{r}<U_{2}\lambda_{0}\leq \sum_{r=1}^{j}\lambda_{r}. \end{array}$$

Since the update step is kept separate from the time and reaction channel sampling steps, we are able to decouple the stochastic simulation algorithm of choice from the spatial considerations of the system. This applies to arbitrary simulation algorithms, including both exact and approximate methods. For example, *τ*-leaping proceeds exactly as described in [19]: the time increment is chosen to satisfy a leap condition, and a Poisson number of events from each reaction channel is chosen to fire. As an example, one might devise a leap condition by restricting the expected number of double-firing events on an individual particle, which necessarily depends on the total number of particles. However, the update step is no longer commutative as updates after a reaction must be carried out sequentially. We cannot sum the total changes to the sample classes in the same fashion as in the well-mixed case, because we must account for the possibility of intersecting particle paths. Thus an additional overhead is needed to randomly shuffle the order in which each reaction channel fires. In the case of exact SSAs, we can use a reaction-reaction dependency graph to restrict the reaction rates that are updated after each event to the subset that is dependent on the fired reaction channel. Unlike their counterparts for CRNs, a dependency between two reactions is captured through common sample classes. Our local updates necessarily affect multiple sample classes which are not uniquely determined by participating particle types as our local update mechanism also affects sample classes. As mentioned earlier, we will follow this manuscript with an extensive review of the many different available well-mixed simulation algorithms applied to IPSs.

## Results

We provide simulation outputs generated by our software for four examples from models of varying complexity. Each demonstrates a phenomenon that is observed in the spatial IPS version of the process but not in the well-mixed CRN version. Jupyter notebooks [32] generating each image can be found at https://github.com/alanderos91/BioSimulator.jl. Animations for each example as well as tutorial notebooks explaining syntax, model construction, and simulation output are also provided through the link. For a list of reactions and parameters for each example, see the S1-S4 Tables in S1 File.

**Fig 3:**
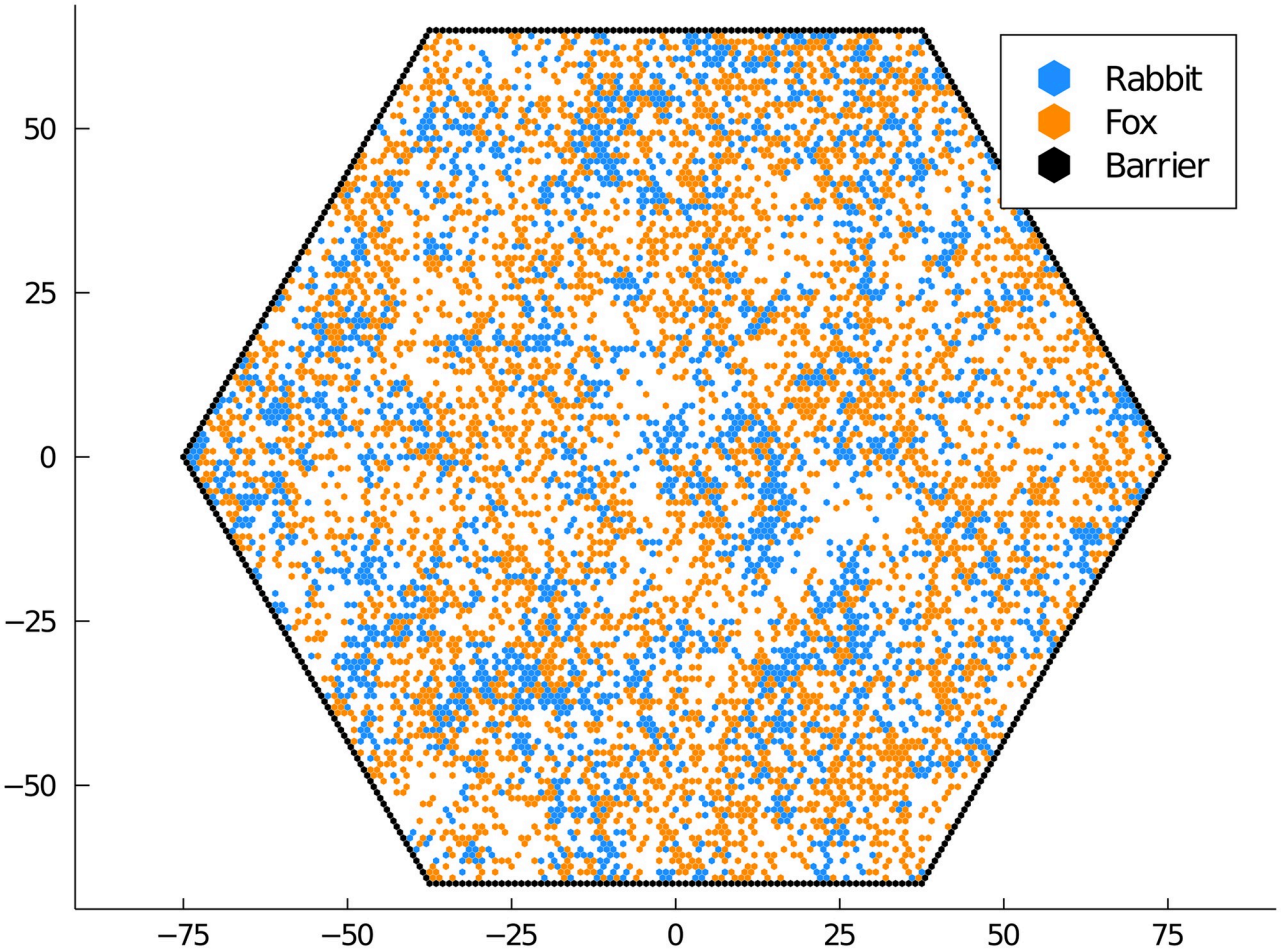
Realization of the predator-prey process. Foxes (predators) and rabbits (prey) diffuse within a bounded domain, undergoing the reactions described in Table 3.

First, we have the predator-prey model previously described in Table 3. The output from the simulation is visualized in Fig 3. An animation of Fig 3 shows spiral wave patterns created by the prey migrating into unoccupied areas while being chased by predators, see the S1 File. The predators at the end of the wave die off, leaving space for the prey to migrate into and repeat the process. In this example, spatial dispersion promotes increased biodiversity. It prevents the large spike in predators that can lead to extinction or dramatic fluctuations in the number of predators and prey commonly seen in the CRN version of the model.

**Fig 4:**
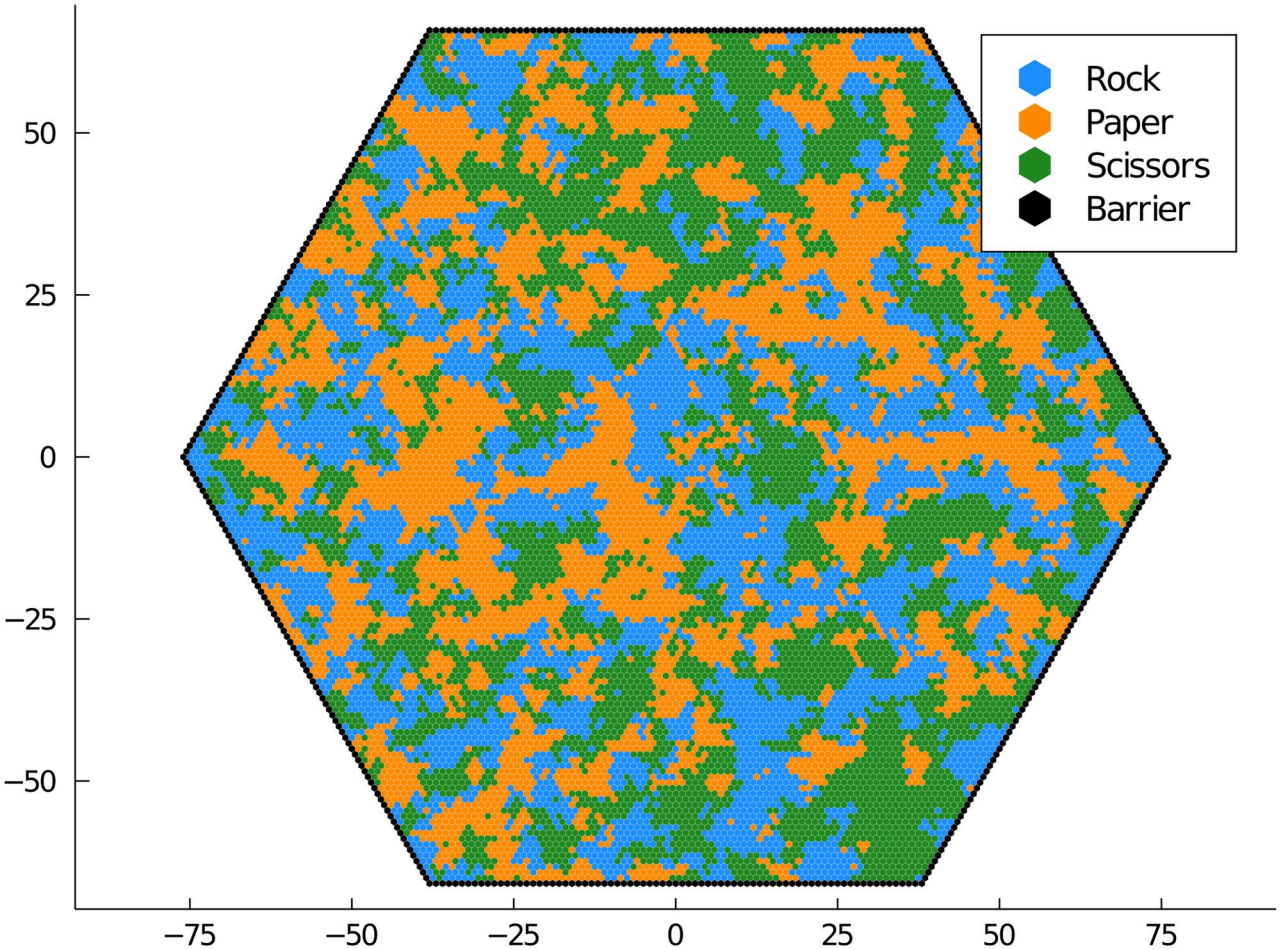
Rock-paper-scissors game. Three different species undergo a birth-death-migration process. Additionally, rock preys on scissors, scissors prey on paper, and paper preys on rock in a cyclic fashion.

Second, we present the three species rock-paper-scissors game depicted in Fig 4. Each species undergoes a birth-death-migration process and has an additional predation reaction: rock preys on scissors, scissors prey on paper, and paper preys on rock. Spiral wave patterns are also observed in this animation. Spatial dispersal similarly maintains biodiversity. Migration at a high rate can destroy this diversity as the populations mix [3].

**Fig 5:**
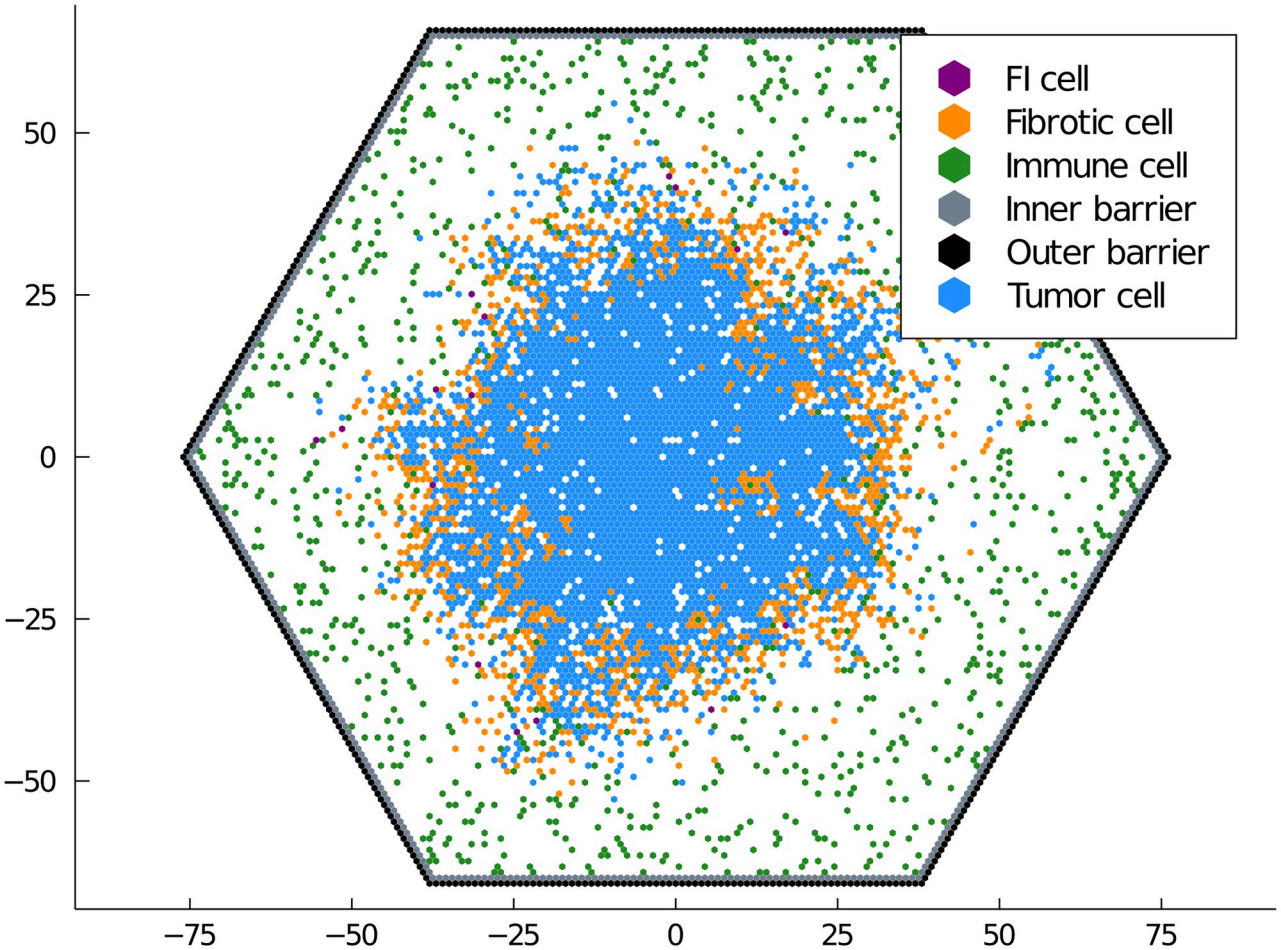
Model of immunotherapy. Tumor cells grow under a birth-death-migration process. Immune cells immigrate from the barrier at constant rate, migrate to cancer cells, then destroy the cancer cells occasionally producing fibrotic cells after this predation. Fibrotic cells are slightly porous to the immune cells but block the diffusion of the cancer cells. FI stands for a fibrotic cell that has an immune cell currently passing through it.

Third, we have a more complicated model of an immune system interacting with a growing tumor in Fig 5. The cancer cells undergo a standard birth-death-migration process. Immune cells migrate in from the barrier cells at a constant rate and destroy tumor cells on contact. This predation may produce a fibrotic cell that is weakly porous to immune cells, simultaneously blocking the spread of the cancer and the eradication of the cancer by the immune system. The formation of a protective shell of fibroblasts is not seen in the well-mixed or RDME cases due to the lack of volume exclusion. Our simulations recapture the immune-excluded response result shown in [28]. This model is useful for exploring potential barriers to tumor eradication during immuno-therapy.

**Fig 6:**
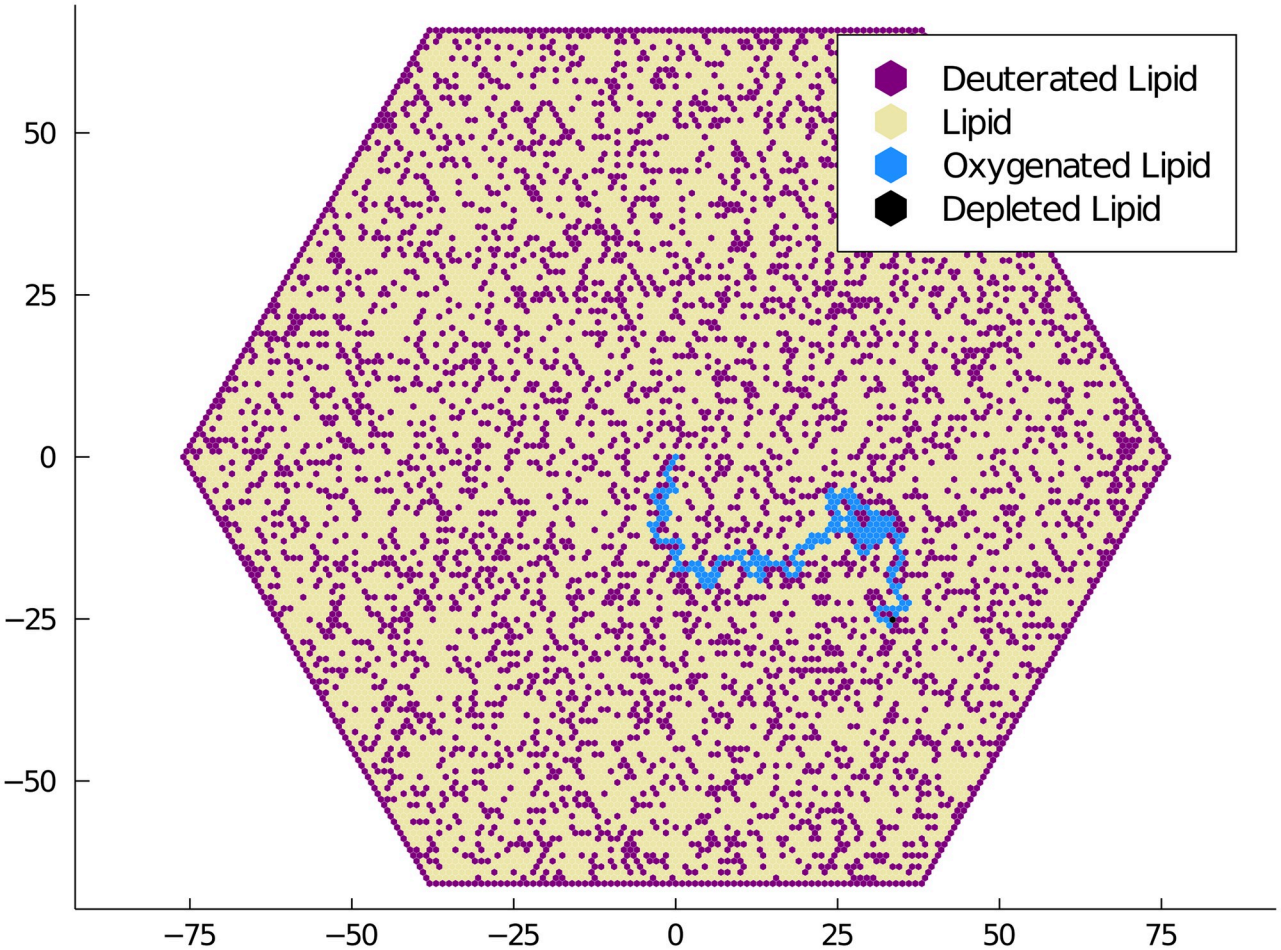
Model of lipid oxidation. Polyunsaturated fatty acids (PUFAs), denoted by open sites, are present in the lipid membranes of cells. Reactive oxygen species can oxygenate a PUFA, resulting in a depleted PUFA that reduces the flexibility of the lipid membrane. Deuterated PUFAs are resistant to the oxygenation. Adding a certain percentage of deuterated PUFAs to the membrane can drastically reduce the length of the depleted lipid chain. The simulation was initiated with a single oxygenated PUFA at the origin.

Finally, we present a model of polyunsaturated fatty acid (PUFA) oxidation in lipid membranes. Certain PUFAs are susceptible to oxidation which creates a kink in their long unsaturated hydrocarbon tails. As a result, a membrane with a significant number of oxidated PUFAs loses flexibility and can lead to neurodegeneration and aging [33]. Replacing the affected hydrogen atoms with deuterium significantly reduces the rate of oxidation, acting as a vaccine of sorts against the infective nature of reactive oxygen species. Fig 6 shows the trail of depleted (kinked) PUFAs left behind a reactive oxygen species jumping to unoxidated PUFAs. There exists a phase transition when the frequency of deuteration reaches approximately a 20%. This transition drastically reduces the length of the depleted PUFA chains left by an oxidated species. This reduction has been observed in vitro through mortality experiments on yeast. It can be observed using our software as a consequence of the oxidated species becoming trapped by its own tail and the deuterated PUFAs.

## Availability and future directions

We have presented a principle for algorithm design that stresses elegance, performance, reproducibility, and wide applicability. These benefits can be broken down into three larger points.

First, our design allows for model standardization based on interacting particle systems. Many *in-silico* studies of spatial particle processes are haphazard in their construction and do not follow continuous time Markovian reaction dynamics. This limits the comparisons that can be made between models and creates barriers for new researchers looking to perform their own simulation studies. Adopting IPSs as a standard mathematical model enhances the most useful application of spatial stochastic simulation, namely generating hypotheses for given phenomena. Having a set of concrete, mechanistic rules with a straightforward probabilistic interpretation allows researchers to develop a hypothesis based on reaction dynamics that reproduce a given behavior *in-silico* and then take these dynamics back to an experimental setting for verification. The PUFA oxidation example serves as a demonstration of how hypotheses about an experimentally observed phenomenon can be tested using our software.

Second, our software is open-source and easily modifiable to individual needs. We have coded our implementation in Julia, a fast, expressive, and flexible open-source programming language designed for scientific computing [25]. Julia’s ease of use facilitates extensions of our software to handle, for example, genealogies, particle tracking, and potentially long-range interactions between particles. The ease of use and model standardization taken together make further research done with our software easily reproducible and straightforward to document.

Lastly, our algorithm design allows us to apply arbitrary well-mixed stochastic simulation algorithms to spatial IPSs. This will be explored later in a review article that compares how each algorithm behaves in the spatial setting. Regardless, we can now apply a large swath of algorithms to spatial stochastic simulation without tedious re-implementation.

It is enlightening to contrast our algorithmic framework with previous work that has been published on different versions of stochastic IPS simulation. The most general spatial approach uses Green’s Function Reaction Dynamics (GFRD) to allow particles to diffuse over a continuous space [34, 35]. This approach does allow for volume exclusion but due to the nature of Brownian motion is numerically intensive. Both Spatiocyte and our approach address this problem by restricting particles to diffuse across a lattice [13, 15, 36]. The software package Spatiocyte is generally similar to what we present here; particles diffuse and react across a lattice obeying volume exclusion. While Spatiocyte is a very sophisticated package with many enhancements, it does not enjoy the advantages of our sample classes in allowing invocation of a range of different stochastic simulation algorithms. Specifically pSpatiocyte, the parallelized and most recent version of Spatiocyte, restricts sampling to Gillespie’s direct method [15].

While we have provided a framework for performing IPS simulations, significant extensions are possible that reduce the bias created by imposing a lattice spatial structure in IPS simulations [14]. First, square lattices are biologically unrealistic and can bias reaction kinetics during simulation. Chew et al. [14] ameliorate this problem imposing a hexagonal close-packed (hcp) lattice. Second, these authors derive a lattice spacing that minimizes the error caused by particles of different sizes. Finally, Chew et al. show how to use a species’ diffusion coefficient to derive diffusion rates on the hcp lattice. Each of these extensions can be implemented in BioSimulator without changing the underlying algorithmic framework. These enhancements and code parallelization along the lines of [15] must await future versions of BioSimulator.

Our implementation of lattice simulation constitutes an extension of the BioSimulator package and is available along with the entire package on the GitHub site https://github.com/alanderos91/BioSimulator.jl. The code can be downloaded anonymously from the GitHub URL. The site includes an issue reporting service as well as documentation, an installation guide, example notebooks, build statuses, and code coverage. BioSimulator is licensed under MIT “Expat” License and is OSI compliant.

## Supporting information

S1 File The file for a complete list of the reactions and parameters used in the examples. We also include animations for each example IPS in the Results sections available through GitHub.
(ZIP)

## References

[1] Waclaw B, Bozic I, Pittman ME, Hruban RH, Vogelstein B, Nowak MA. A spatial model predicts that dispersal and cell turnover limit intratumour heterogeneity. Nature. 2015;525(7568):261. 10.1038/nature14971 26308893 PMC4782800

[2] Kéfi S, Rietkerk M, Alados CL, Pueyo Y, Papanastasis VP, ElAich A, et al. Spatial vegetation patterns and imminent desertification in Mediterranean arid ecosystems. Nature. 2007;449(7159):213. 10.1038/nature06111 17851524

[3] Reichenbach T, Mobilia M, Frey E. Mobility promotes and jeopardizes biodiversity in rock–paper–scissors games. Nature. 2007;448(7157):1046. 10.1038/nature06095 17728757

[4] Asmussen S, Glynn PW. Stochastic simulation: algorithms and analysis. vol. 57. Springer Science & Business Media; 2007.

[5] Gillespie DT, Hellander A, Petzold LR. Perspective: Stochastic algorithms for chemical kinetics. The Journal of Chemical Physics. 2013;138(17):05B201_1. 10.1063/1.4801941 23656106 PMC3656953

[6] Grima R, Schnell S. A systematic investigation of the rate laws valid in intracellular environments. Biophysical chemistry. 2006;124(1):1–10. 10.1016/j.bpc.2006.04.019 16781049

[7] Schnell S, Turner T. Reaction kinetics in intracellular environments with macromolecular crowding: simulations and rate laws. Progress in biophysics and molecular biology. 2004;85(2-3):235–260. 10.1016/j.pbiomolbio.2004.01.012 15142746

[8] Liggett TM. Interacting particle systems. vol. 276. Springer Science & Business Media; 2012.

[9] Cianci C, Smith S, Grima R. Molecular finite-size effects in stochastic models of equilibrium chemical systems. The Journal of chemical physics. 2016;144(8):084101. 10.1063/1.4941583 26931675

[10] Cianci C, Smith S, Grima R. Capturing Brownian dynamics with an on-lattice model of hard-sphere diffusion. Physical Review E. 2017;95(5):052118. 10.1103/PhysRevE.95.052118 28618561

[11] Smith S, Grima R. Spatial stochastic intracellular kinetics: A review of modelling approaches. Bulletin of mathematical biology. 2019;81(8):2960–3009. 10.1007/s11538-018-0443-1 29785521 PMC6677717

[12] Liggett TM. Stochastic interacting systems: contact, voter and exclusion processes. vol. 324. Springer Science & Business Media; 2013.

[13] Arjunan SN, Takahashi K. Multi-algorithm particle simulations with Spatiocyte. In: Protein Function Prediction. Springer; 2017. p. 219–236. 10.1007/978-1-4939-7015-5_16 28451982

[14] Chew WX, Kaizu K, Watabe M, Muniandy SV, Takahashi K, Arjunan SN. Surface reaction-diffusion kinetics on lattice at the microscopic scale. Physical Review E. 2019;99(4):042411. 10.1103/PhysRevE.99.042411 31108654

[15] Arjunan SN, Miyauchi A, Iwamoto K, Takahashi K. pSpatiocyte: a high-performance simulator for intracellular reaction-diffusion systems. BMC bioinformatics. 2020;21(1):1–21. 10.1186/s12859-019-3338-8 31996129 PMC6990473

[16] Bortz AB, Kalos MH, Lebowitz JL. A new algorithm for Monte Carlo simulation of Ising spin systems. Journal of Computational Physics. 1975;17(1):10–18. 10.1016/0021-9991(75)90060-1

[17] Gillespie DT. Exact stochastic simulation of coupled chemical reactions. The Journal of Physical Chemistry. 1977;81(25):2340–2361. 10.1021/j100540a008

[18] Gillespie DT. Approximate accelerated stochastic simulation of chemically reacting systems. The Journal of Chemical Physics. 2001;115(4):1716–1733. 10.1063/1.1378322

[19] Cao Y, Gillespie DT, Petzold LR. Efficient step size selection for the tau-leaping simulation method. The Journal of Chemical Physics. 2006;124(4):044109. 10.1063/1.2159468 16460151

[20] Sehl M, Alekseyenko AV, Lange KL. Accurate stochastic simulation via the step anticipation *τ*-leaping (SAL) algorithm. Journal of Computational Biology. 2009;16(9):1195–1208. 10.1089/cmb.2008.0249 19772431 PMC3148118

[21] Marquez-Lago TT, Burrage K. Binomial tau-leap spatial stochastic simulation algorithm for applications in chemical kinetics. The Journal of Chemical Physics. 2007;127(10):09B603. 10.1063/1.2771548 17867731

[22] Anderson DF. Incorporating postleap checks in tau-leaping. The Journal of Chemical Physics. 2008;128(5):054103. 10.1063/1.2819665 18266441

[23] Auger A, Chatelain P, Koumoutsakos P. R-leaping: Accelerating the stochastic simulation algorithm by reaction leaps. The Journal of Chemical Physics. 2006;125(8):084103. 10.1063/1.2218339 16964997

[24] Landeros A, Stutz TC, Keys KL, Alekseyenko A, Sinsheimer JS, Lange K, et al. BioSimulator. jl: Stochastic simulation in Julia. Computer Methods and Programs in Biomedicine. 2018;167:23–35. 10.1016/j.cmpb.2018.09.009 30501857 PMC6388686

[25] Bezanson J, Edelman A, Karpinski S, Shah VB. Julia: A fresh approach to numerical computing. SIAM Review. 2017;59(1):65–98. 10.1137/141000671

[26] Gibson MA, Bruck J. Efficient Exact Stochastic Simulation of Chemical Systems with Many Species and Many Channels. The Journal of Physical Chemistry A. 2000;104(9):1876–1889. 10.1021/jp993732q

[27] McCollum JM, Peterson GD, Cox CD, Simpson ML, Samatova NF. The sorting direct method for stochastic simulation of biochemical systems with varying reaction execution behavior. Computational Biology and Chemistry. 2006;30(1):39–49. 10.1016/j.compbiolchem.2005.10.007 16321569

[28] Kather JN, Poleszczuk J, Suarez-Carmona M, Krisam J, Charoentong P, Valous NA, et al. In silico modeling of immunotherapy and stroma-targeting therapies in human colorectal cancer. Cancer Research. 2017;77(22):6442–6452. 10.1158/0008-5472.CAN-17-2006 28923860

[29] Lange K. Applied probability. Springer Science & Business Media; 2010.

[30] Chatterjee A, Vlachos DG. An overview of spatial microscopic and accelerated kinetic Monte Carlo methods. Journal of Computer-aided Materials Design. 2007;14(2):253–308. 10.1007/s10820-006-9042-9

[31] Nijenhuis A, Wilf HS. Combinatorial algorithms: for computers and calculators. Elsevier; 2014.

[32] Perkel JM. Why Jupyter is data scientists’ computational notebook of choice. Nature. 2018;563(7732):145–147. 10.1038/d41586-018-07196-1 30375502

[33] Firsov AM, Fomich MA, Bekish AV, Sharko OL, Kotova EA, Saal HJ, et al. Threshold protective effect of deuterated polyunsaturated fatty acids on peroxidation of lipid bilayers. The FEBS Journal. 2019;286(11):2099–2117. 10.1111/febs.14807 30851224

[34] van Zon JS, Ten Wolde PR. Green’s-function reaction dynamics: a particle-based approach for simulating biochemical networks in time and space. The Journal of Chemical Physics. 2005;123(23):234910. 10.1063/1.2137716 16392952

[35] Sokolowski TR, Paijmans J, Bossen L, Miedema T, Wehrens M, Becker NB, et al. eGFRD in all dimensions. The Journal of Chemical Physics. 2019;150(5):054108. 10.1063/1.5064867 30736681

[36] Arjunan S, Tomita M. A new multicompartmental reaction-diffusion modeling method links transient membrane attachment of E. coli MinE to E-ring formation. Nature Precedings. 2009; p. 1–1. 10.1007/s11693-009-9047-2 20012222 PMC2816228

