# Supplementary material for: Stochastic simulation algorithms for Interacting Particle Systems
Source: PLoS One. 2021 Mar 2;16(3):e0247046. doi: 10.1371/journal.pone.0247046 (PMC7924777; doi:10.1371/journal.pone.0247046)
Supplement: S1 File — We also include animations for each example IPS in the Results sections available through GitHub. (ZIP) [file pone.0247046.s001.zip › Revised Supplementary Information.pdf]

Stochastic Simulation Algorithms for Interacting Particle Systems

Supplementary Information

Timothy C Stutz,[1] Alfonso Landeros,[1] Jason Xu,[2] Janet S Sinsheimer,[1,3,4] Mary Sehl,[1,5] and Kenneth Lange[1,3,6]

[1)] *Department of Computational Medicine, University of California, Los Angeles CA 90095*

[2)] *Department of Statistical Sciences, Duke University, Durham NC 27708*

[3)] *Department of Human Genetics, David Geffen School of Medicine, University of California, Los Angeles CA 90095*

[4)] *Department of Biostatistics, UCLA Fielding School of Public Health, Los Angeles CA 90095*

[5)] *Division of Hematology-Oncology, Department of Medicine, David Geffen School of Medicine, University of California, Los Angeles CA 90095*

[6)] *Department of Statistics, University of California, Los Angeles CA 90095*

(Dated: 4 December 2020)

Section	Time	% Total Run-Time
Sampling the next reaction to fire	319ms	1.49%
Reaction Execution:	20.8s	97.0%
Sampling particles to undergo the reaction	1.81s	8.70%
Updating sample classes and particle types	18.2s	87.5%
Updating the reaction rates	318ms	1.48%

S1 Table: Breakdown of the simulation run-time for the predator-prey example, Fig 3. The Reaction Execution step contains the two sub-steps indented below. Executing the sampled reaction consists of the bulk of the simulation time, particularly the steps where we update the sample classes and particle types of the particles involved and adjacent to each reaction. In comparison the stochastic simulation algorithm portions of the run-time are minimal.

Name	Diagram	Rate parameter
Fox Predation	$F+R \rightarrow F+0$	0.35
Fox Migration	$F+0 \rightarrow 0+F$	1.0
Fox Death	$F \rightarrow 0$	0.3
Rabbit Reproduction	$R+0 \rightarrow R+R$	0.2
Rabbit Migration	$R+0 \rightarrow 0+R$	1.0
Rabbit Death	$R \rightarrow 0$	0.1

S2 Table: Reaction channels and parameter values for the predator-prey example simulation, Fig 3. F denotes a fox (predator), R denotes a rabbit (prey), 0 denotes an empty site. All rates have units 1/time.

Name	Diagram	Rate parameter
Rock Predation	$R+S \rightarrow R+R$	2.0
Rock Migration	$R+0 \rightarrow 0+R$	1.0
Rock Death	$R \rightarrow 0$	0.1
Rock Reproduction	$R+0 \rightarrow R+R$	0.2
Paper Predation	$P+R \rightarrow P+P$	2.0
Paper Migration	$P+0 \rightarrow 0+P$	1.0
Paper Death	$P \rightarrow 0$	0.0
Paper Reproduction	$P+0 \rightarrow P+P$	0.1
Scissors Predation	$S+P \rightarrow S+S$	2.0
Scissors Migration	$S+0 \rightarrow 0+S$	1.0
Scissors Death	$S \rightarrow 0$	0.0
Scissors Reproduction	$S+0 \rightarrow S+S$	0.1

S3 Table: Reaction channels and parameter values for the rock-paper-scissors example simulation, Fig 4. R denotes a rock, P denotes a paper, R denotes a rabbit (prey), and 0 denotes an empty site. All rates have units 1/time.

Name	Diagram	Rate parameter
Tumor Cell Migration	$T+0 \to 0+T$	0.5
Immune Cell Migration	$I+0 \to 0+I$	5.0
Tumor Cell Reproduction	$T+0 \to T+T$	0.03
Immune Cell Predation	$I+T \to I+0$	0.2
Immune Cell Recruitment	$I+T \to I+I$	2×10^{-7}
Immune Cell Immigration	$B+0 \to B+I$	0.02
Tumor Cell Death	$T \to 0$	0.01
Immune Cell Death	$I \to 0$	0.01
Immune Migration through Fibrotic Cell	$F+I \to FI+0$	0.02
Immune Migration through Fibrotic Cell	$FI+0 \to F+I$	0.02
Immune Predation through Fibrotic Cell	$FI+T \to F+I$	0.2
Fibrotic Cell Production	$I+T \to I+F$	0.2

S4 Table: Reaction channels and parameter values for the immunotherapy example simulation, Fig 5. T denotes a tumor cell, I denotes an immune cell, B denotes a barrier cell that can produce immune cells, F denotes a fibrotic cell, and FI denotes a fibrotic cell being passed through by an immune cell. All rates have units 1/time.

Name	Diagram	Rate parameter
Lipid Oxidation	$OL+L \to DL+OL$	1.0

S5 Table: Reaction channels and parameter values for the polyunsaturated fatty acid oxidation example simulation, Fig 6. L denotes a lipid, OL a lipid with a reactive oxygen species, and DL denotes a depleted lipid that has been oxidized. All rates have units 1/time.
